# Significant improvements in cataract treatment and persistent inequalities in access to cataract surgery among older Poles from 2009 to 2019: results of the PolSenior and PolSenior2 surveys

**DOI:** 10.3389/fpubh.2023.1201689

**Published:** 2023-10-10

**Authors:** Natalia Lange, Hanna Kujawska-Danecka, Adam Wyszomirski, Klaudia Suligowska, Adrian Lange, Dorota Raczyńska, Justyna Jędrychowska-Jamborska, Małgorzata Mossakowska

**Affiliations:** ^1^Department of Preventive Medicine and Education, Medical University of Gdańsk, Gdańsk, Poland; ^2^Department of Internal Medicine, Connective Tissue Diseases and Geriatrics, Medical University of Gdańsk, Gdańsk, Poland; ^3^Department of Adult Neurology, Faculty of Medicine Medical, University of Gdańsk, Gdańsk, Poland; ^4^Department of Dental Techniques and Masticatory System Dysfunctions, Medical University of Gdańsk, Gdańsk, Poland; ^5^Optimum Professor's Ophthalmology Centre, Gdańsk, Poland; ^6^Clinical Unit, Department of Ophthalmology, Wojewódzki Hospital in Cracow, Cracow, Poland; ^7^Aging and Longevity Strategic Project, International Institute of Molecular and Cell Biology, Warsaw, Poland

**Keywords:** cataract surgery, health inequities, gender, socioeconomic factors, aged, population characteristics, PolSenior, PolSenior2

## Abstract

**Background and aims:**

Cataract is the leading cause of visual impairment and blindness among older adults worldwide, that can be corrected through surgical interventions. However, diagnosis and treatment bias can be observed, and it is a major issue for improving health policies. Therefore, we assessed a declared prevalence of cataract and the frequency of surgical treatment of this condition in the Polish population in the years 2009–2019. To provide evidence of health inequalities, we compared operated and non-operated seniors using selected socioeconomic factors and identified variables affecting the availability of cataract surgery services over a 10-year follow-up period.

**Methods:**

An analysis based on a survey among 4,905 participants of the nationwide PolSenior study conducted in 2008–2009, and 5,031 participants of PolSenior2 conducted one decade later to assess the health of Poles over 65 years of age.

**Results:**

Cataract diagnosis was declared by 25.5 and 28.2% of the study population in PolSenior and PolSenior2 surveys, respectively. Out of those diagnosed with cataract in PolSenior, 46.5% underwent surgical treatment for at least one eye. This rate increased up to 67.9% in the survey conducted 10 years later. Independent factors increasing the chance for cataract surgery in both cohorts included male sex and age > 75 years. Additional factors were self-reported good health status in PolSenior and lack of financial problems in purchasing medicines in PolSenior2. Over the investigated decade, the chances for cataract surgical treatment increased in single-living and widowed patients. The shortage of funds for medications remained the only significant barrier for surgery.

**Conclusion:**

Although the prevalence of cataract in the older adult population in Poland has not changed from 2009 to 2019, the rate of cataract surgeries has considerably increased over the analyzed decade. Patients with lower socioeconomic status and women have lower access to surgical cataract management.

## 1. Introduction

Cataract remains the leading cause of blindness worldwide in people ≥50 years of age, accounting for about 45% of global blindness cases and affecting 15.2 million people in 2020. It is also the second leading cause of moderate to severe vision impairment (78.8 million) following uncorrected refractive error (86.1 million) (1). In addition to decreased visual acuity, a patient with cataracts may experience other symptoms such as reduced contrast sensitivity, impaired color vision, double vision, glare, and ‘halos’ around lights. This condition most often affects older adults as a result of biological aging (age-related cataracts). Cataract is associated with decreased quality of life and reduced life expectancy (1–3).

Although cataract-induced blindness is particularly common in developing countries, together with age-related macular degeneration (AMD), it is still a major public health problem in developed countries (1) including Poland. Poland has a population of 37.6 million and is located in Central and Eastern Europe. However, there is only few published data on the prevalence of cataract in this region (4).

Phacoemulsification combined with intraocular lens implantation is the most common technique for cataract removal both globally and in Poland (5, 6). Treatment rates have been growing successively over the last few years (5, 6), however the issue of inequalities in the distribution of cataract surgery services remains open. There is abundant evidence that social factors, including education, employment status, income level and gender have significant impact on health status (7–10) and accessibility of the health care system regarding diagnostic and treatment in equal measure. The socio-demographic status of the Polish population is becoming diverse, which can cause unfair inequalities in health (11).

Determining the prevalence of cataracts in a representative sample of Poles and identifying groups whose health care is inadequate in this regard would create grounds for active and effective prevention of serious consequences of cataracts including blindness. Identification of subgroups with untreated disease would enable early educational, preventive and therapeutic intervention.

The aim of the study was to assess changes in the prevalence of cataract and surgical treatment rates considering selected socio-economic factors in a representative sample of Poles aged 65 years and older over a decade, based on the PolSenior (12) and PolSenior2 (13) studies.

## 2. Materials and methods

### 2.1. Study sample and procedures

PolSenior, a cross-sectional study conducted between 2008 and 2009 in a representative sample of Polish adults aged 65 years and over, comprised 4,979 participants, and has been acknowledged as the most important project monitoring the health of Polish seniors, including eyesight screening (12). After a decade, in 2018–2019, the survey was repeated as part of the PolSenior2 study in a representative sample of 5,987 adults aged 60 years and over (13). In both studies, participants were recruited from all administrative regions in Poland using a three-stage stratified, proportional draw, in 5 years old cohorts. Cohorts were similar in number and consisted of similar numbers of women and men. The representativeness of the sample was obtained by weighting for structure of the older Polish population (12–14). In PolSenior2 project a new cohort was drawn (13), therefore two study populations formed disjoint sets.

Details of sample selection, methods and study design in a randomly selected representative sample of old Poles in PolSenior projects were described in previous publications (12, 13).

The analyses presented in this publication include comparable groups from both studies, 4,979 subjects aged 65 and older from PolSenior and 5,056 subjects of comparable age from PolSenior2. The group of 930 subject aged 60–64 representing PolSenior2 was not included in the analyses.

A small percentage of respondents was removed as they did not provide answers concerning cataract in the survey. A total of 4,905 (2,534 men and 2,371 women, 98.5%) participants responded to the question on cataract diagnosis in PolSenior compared to 5,031 (99.5%) respondents (2,482 men and 2,549 women) in PolSenior2. Of the 1,419 respondents declaring cataract diagnosis in PolSenior, 91.5% (595 men and 703 women) answered the question about cataract treatment. In PolSenior2, cataract was found in 1,715 participants, of whom 95.1% (698 men, 933 women) reported treatment.

This paper compares the findings on the prevalence of cataract, age at diagnosis and surgical treatment in two populations of Polish seniors. The data were analysed by age group (65–74, 75–84, ≥85 years), sex, educational level and place of residence. The impact of socioeconomic factors on cataract surgery rates was also assessed and the intercohorts relationships between PolSenior and PolSenior2 were analysed. Additionally, the analysis included marital status, living status, and self-rated health (SRH).

### 2.2. Survey procedures

The protocol of the PolSenior2 study was based on the protocol of the PolSenior study and the same questions were included in the analysis. The study protocol consisted of three paper-version questionnaires. Medical and socioeconomic surveys were face-to-face interviews performed by trained nurses during three visits at participants homes. Some data were collected by a self-completion questionnaire, filled in individually by respondents. In the Table 1 we attached a detailed list of questions used for the purpose of the present study.

**Table 1:** The list of questions and response options included in questionnaires, used for the purpose of the study.

Question	Response options	Comments
Has your doctor ever diagnosed you with cataracts?	Yes No	
How old were you when your doctor first diagnosed you with cataracts?	(age)	
Was the disease…. (treatment)	Operated Unoperated I do not remember/ know	If only one eye was operated, the answer “operated” was marked.
What is your education? (education level)	Primary or incomplete primary Basic vocational or gymnasium Middle secondary and post secondary Higher	
How would you describe your current personal situation? (marital status)	Bachelor/ maiden Married Widower/widow Divorced/living in separation	
Do any other people live in the apartment/house with you? Whom do you live with?” (living status)	Yes/no Husband/wife (including former), partner(s), Children / grandchildren/ great-grandchildren, parents, parents-in-law Other family members, people outside the family	If No Living alone If Yes: with spouse only with other people
How many years in total did you work professionally?	(number of years)	
Which of the following sentences best describes the financial situation in your household? (financial situation of the household)	I/we can afford everything I/we can afford for most thing when saving I/we have difficulties with paying for food or clothes	
Have you run out of money to buy medications in the past 12 months?	Yes (sometimes or often) No	
Please indicate how you assess your current state of health (on a scale of 0 to 10), assuming that 0 is the worst state of health imaginable, while 10 is the best state of health imaginable.	(number between 0 to 10 on *Visual Analogue Scale*)	0–3 – classified as having a poor self-reported health status 4–6 – fair self-reported health status 7–10 good. Self-reported health status.

### 2.3. Statistical analyses

Continuous variables are presented as means with standard deviations or medians with interquartile ranges. Differences between the groups were verified with the Student’s t-test or the Mann–Whitney U-test. Categorical data is presented as counts and percentages. The chi-square test or Fisher’s exact test was used to compare the groups. The logistic regression method was applied to assess the relationship between cataract treatment and the set of independent variables. Additional models analysed interaction to assess changes over time, i.e., between the PolSenior and PolSenior2 cohorts. Multivariate models were developed using the backward stepwise procedure, including only cases with a complete set of data. Regression coefficients were used to calculate odds ratios (ORs) with 95% confidence intervals (95% CI). Statistical results were considered significant at *p* ≤ 0.05.

The results in Tables 2, 3 are presented after the weighting procedures for the Polish population as percentage or mean values with 95% CI. A statistically significant difference between the groups was assumed for non-overlapping confidence intervals. Statistical packages R (R Core Team, version 3.6.3) and SAS 9.4 TS Level 1 M5 were used for the analysis.

**Table 2:** The frequency of declared cataract diagnosis in the population of Polish seniors depending on age, education, place of residence based on PolSenior and PolSenior2.

	Cataract					
	Diagnosed - PolSenior			Diagnosed - PolSenior2		
	Males	Females	Total	Males	Females	Total
	% (95% CI)	% (95% CI)	% (95% CI)	% (95% CI)	% (95% CI)	% (95% CI)
Age group [years]						
Total 65 and more	21.3 (17.9–24.6)	28.0 (24.2–31.9)	25.5 (22.6–28.4)	20.6 (18.6–22.6)	33.2 (30.5–35.8)	28.2 (26.3–30.1)
65–74	15.9 (10.3–21.5)	21.9 (17.1–26.8)	19.4 (15.5–23.3)	12.6 (10.2–15.1)	21.2 (17.9–24.6)	17.5 (15.4–19.6)
75–84	26.1 (22.2–30.0)	32.9 (28.1–37.6)	30.5 (27.1–33.9)	31.4 (27.3–35.4)	43.4 (38.8–47.9)	39.0 (35.6–42.5)
85+	41.7 (37.2–46.2)	37.3 (30.3–44.4)	38.5 (33.3–43.8)	49.4 (43.4–55.4)	58.5 (53.6–63.5)	56.0 (51.7–60.2)
Education						
Primary or incomplete primary	20.0 (15.9–24.1)	25.2 (20.5–29.9)	23.7 (20.2–27.1)	22.6 (18.0–27.3)	37.6 (33.6–41.6)	32.8 (29.5–36.2)
Basic vocational	15.8 (10.2–21.4)	20.5 (12.9–28.2)	17.6 (13.0–22.3)	17.8 (14.7–21.0)	28.2 (21.6–34.8)	22.5 (19.0–26.0)
Middle, secondary or post-secondary	27.3 (18.2–36.4)	36.1 (29.9–42.3)	33.0 (28.4–37.6)	21.4 (17.7–25.2)	32.4 (27.1–37.8)	28.4 (24.6–32.3)
Higher	23.3 (13.8–32.8)	26.5 (15.0–38.1)	24.7 (17.2–32.2)	22.5 (16.8–28.1)	28.2 (21.7–34.7)	25.9 (21.7–30.1)
Place of residence [number of inhabitants]						
Rural	16.9 (13.1–20.7)	20.3 (14.9–25.8)	19.2 (15.4–23.0)	15.8 (12.3–19.3)	28.5 (24.9–32.1)	23.5 (20.7–26.2)
Urban < 50,000	17.1 (12.5–21.8)	26.7 (22.0–31.5)	23.0 (19.5–26.4)	21.0 (16.7–25.4)	32.7 (27.9–37.5)	28.3 (25.2–31.4)
Urban 50–200,000	23.4 (18.0–28.8)	30.6 (22.8–38.5)	27.7 (22.3–33.2)	24.1 (19.9–28.4)	38.1 (31.4–44.7)	31.9 (27.7–36.1)
Urban > 200,000	28.0 (18.7–37.3)	39.6 (32.9–46.3)	34.8 (29.0–40.6)	26.1 (20.5–31.7)	38.4 (32.7–44.0)	33.8 (29.2–38.4)

CI, confidence interval.

**Table 3:** Cataract surgery rates (in the subgroup of patients with cataract) in the population of Polish seniors by age, education, place of residence, based on PolSenior and PolSenior2.

	Cataract					
	Operated – PolSenior			Operated – PolSenior2		
	Males	Females	Total	Males	Females	Total
	% (95% CI)	% (95% CI)	% (95% CI)	% (95% CI)	% (95% CI)	% (95% CI)
Age group [years]						
Total 65 and more	54.6 (47.5–61.7)	42.8 (37.9–47.6)	46.5 (41.8–51.3)	75.4 (70.7–80.1)	64.9 (61.3–68.6)	67.9 (64.9–71)
65–74	59.9 (45.1–74.6)	34.0 (25.4–42.5)	42.8 (34.9–50.8)	74.5 (65.5–83.6)	58.2 (50.4–66.0)	63.2 (57.5–69)
75–84	46.1 (38.8–53.3)	49.7 (41.2–58.2)	48.6 (42.0–55.2)	74.9 (68.1–81.6)	66.0 (59.5–72.5)	68.6 (63.4–73.8)
85+	64.1 (54.6–73.6)	45.0 (35.9–54.0)	50.4 (43.3–57.5)	78.2 (70.1–86.3)	72.9 (67.6–78.2)	74.2 (69.5–79.0)
Education						
Primary or incomplete primary	51.0 (39.2–62.7)	42.4 (34.5–50.3)	44.5 (38.1–51.0)	79.1 (70.9–87.3)	61.9 (55.9–67.9)	65.5 (59.9–71.1)
Basic vocational	53.7 (41.5–65.8)	44.7 (24.3–65.1)	49.7 (37.3–62.0)	75.9 (67.8–84)	65.8 (55.7–75.8)	70.2 (63.6–76.8)
Middle, secondary or post-secondary	71.3 (57.3–85.3)	43.5 (32.7–54.3)	51.5 (41.1–61.9)	72.3 (62.3–82.3)	65.9 (59.8–72.1)	67.7 (62.6–72.9)
Higher	33.8 (12.4–55.2)	40.5 (23.3–57.8)	36.7 (20.9–52.6)	76.6 (63.6–89.6)	69.7 (59.8–79.7)	72.1 (63.8–80.4)
Place of residence [number of inhabitants]						
Rural	51.3 (42.8–59.9)	42.2 (33.7–50.6)	44.8 (37.8–51.8)	74.9 (66.7–83.0)	63.8 (56.9–70,8)	66.7 (60.9–72.5)
Urban < 50,000	52.1 (39.4–64.7)	40.4 (30.3–50.5)	43.8 (35.3–52.4)	77.5 (71.1–84.0)	64.0 (56.2–71.8)	67.7 (62.3–73.0)
Urban 50–200,000	43.4 (30.6–56.2)	44.0 (32.6–55.4)	43.8 (33.6–54.0)	78.6 (71.5–85.8)	70.7 (62.1–79.4)	73.5 (67.5–79.5)
Urban > 200,000	62.6 (48.5–76.7)	44.0 (34.9–53.2)	50.4 (40.7–60.0)	71.4 (58.2–84.5)	63.1 (56.8–69.4)	65.5 (59.1–71.9)

CI, confidence interval.

## 3. Results

### 3.1. The prevalence of cataract

Cataract diagnosis was declared in the PolSenior project by 25.5% of the Polish population aged ≥65 years. There were no significant differences between women and men (Table 2). The prevalence of cataract increased with age in both sexes. Cataract was reported by urban (>200,000 inhabitants) respondents more often than by rural residents (34.8 vs. 19.2%). In PolSenior2, a history of cataract was reported by 28.2% of the study population, more often by women (33.2 vs. 20.6%) – Table 2. The prevalence increased with age and was higher in city dwellers (>50,000 inhabitants) as compared to rural residents. Although cataract was declared by a comparable percentage of the older population in both editions of PolSenior, the awareness of the disease increased over the decade among women aged ≥75 years and among women with at most primary education (Table 2). There were no significant changes in the prevalence of cataract over the decade among men. In PolSenior, cataract was diagnosed on average 2 years later than in PolSenior2 (74.2 years vs. 72.1 years, *p* < 0.005; Table 4).

**Table 4:** Age at the time of cataract diagnosis in the population of Polish seniors, based on PolSenior and PolSenior2.

	PolSenior1 (K vs. M, *p* = 0,002)			PolSenior2 (K vs. M, p = 0,063)		
	Females	Males	Total	Females	Males	Total
Mean	73,7	74,9	74,2	71,7	72,6	72,1
Median (Q1,Q3)	74 (68,81)	76 (70,82)	75 (69,82)	73 (67,79)	74 (68,80)	73 (67,80)

Unweighted data.

It was observed that at the time of diagnosis women were on average 1 year younger than men, which was statistically significant only in the PolSenior study (women – 73.7 years; men – 74.9 years, *p* = 0.002). The age data presented at diagnosis were not weighted (Table 4).

### 3.2. Surgical treatment

In PolSenior, 46.5% of respondents diagnosed with cataract had at least one eye treated surgically (Table 4). Surgical treatment was more common in men than in women in the youngest (65–74 years) and oldest age groups (≥85 years), but for the total older population, the difference between the sexes did not reach statistical significance. The percentage of operated respondents did not differ significantly in individual age groups, and it did not depend on the level of education or place of residence (Table 3).

A decade later, cataract surgery was declared by significantly more (67.9%) seniors (Table 3), with male predominance (75.4 vs. 64.9%). The percentage of respondents with a history of cataract surgery was higher in older age groups, but did not depend on the level of education or place of residence.

A significant increase in the percentage of operated patients was observed among women in each of the study age groups, and among men in the 75–84 year subgroup (Table 4). Although cataract surgery rates increased among respondents at all education levels, the largest, two-fold difference was found among those with higher education (36.7 vs. 72.1%). Cataract surgery rates among the inhabitants of rural and urban (up to 200,000 inhabitants) regions were found to increase about 1.5-fold over the decade. For respondents in big cities of >200,000 inhabitants, a significant increase was observed only in the female group (44.0 vs. 63.1%).

### 3.3. Comparison of operated vs. non-operated PolSenior and PolSenior2 respondents in regard to selected socioeconomic factors

Supplementary Table 1 compares groups of seniors reporting cataract diagnosis who were or were not operated in relation to selected socioeconomic factors. Age and sex were the only variables that significantly differentiated respondents with operated and unoperated cataract in both PolSenior editions. Non-surgical patients were younger. Surgeries were more common in men. Additionally, among the analysed variables in PolSenior, the group of operated/non-operated patients also differed in terms of marital and living status, SRH and financial situation. In PolSenior2, surgical respondents had longer work histories, and were less likely to report lack of funds for medications. Other investigated factors, including the place of residence (rural/urban), level of education, type of work performed in the past, frequency of general practitioner (GP) visits, or the need for regular assistance, were not related to cataract surgery rates in either of the two studies.

### 3.4. Univariate and multivariate logistic regression analysis

All variables associated with cataract surgery in PolSenior or PolSenior2 were included in the regression analysis. The results for both study cohorts and the changes between the cohorts are presented in Table 5. In both cohorts, age over 75 years and male sex were correlated with higher surgical rates. Men were 1.7 times (95% confidence interval [CI]: (1.359–2.118); *p* < 0.001) more likely to have cataract surgery than women in 2009, and 1.5 times [95%CI, (1.223–1.913); *p* < 0.001] in 2019. In PolSenior, being in a relationship, living with a spouse only, good financial situation and good SRH were additional factors improving access to cataract treatment. On the other hand, in PolSenior2, apart from female sex and age 65–75, the declared lack of funds for medications was the only significant factor related to the failure to undergo cataract surgery. A multivariate analysis (Table 6) confirmed that male sex and age > 75 years were independent factors promoting cataract surgery in both cohorts. Moreover, good SRH and no financial difficulties in purchasing medications were additional factors related to surgical treatment in PolSenior and PolSenior2, respectively.

**Table 5:** Univariate regression analysis of relationship between catarct treatment and the set of independent variables in PolSenior and PolSenior2 cohort.

		PolSenior cohort OR (95%CI); *p*-value	PolSenior 2 cohort OR (95%CI); *p*-value	Cohort × Predictor interaction OR (95%CI); *p*-value
Predictors	Categories			
Sex	Males	1.697 (1.359–2.118);**<0.001**	1.530 (1.223–1.913);**<0.001**	0.902 (0.658–1.236); 0.519
	Females	Ref.	Ref.	Ref.
Age	>75	1.499 (1.165–1.929);**0.002**	1.610 (1.274–2.034);**<0.001**	1.074 (0.761–1.514); 0.683
	65–75	Ref.	Ref.	Ref.
Marital status	Unmarried/Divorced/Separated	0.744 (0.392–1.409); 0.364	0.947 (0.561–1.598); 0.838	1.273 (0.562–2.947); 0.567
	Widowed	0.715 (0.568–0.898);**0.004**	1.076 (0.858–1.349); 0.526	1.506 (1.092–2.078);**0.013**
	Married	Ref.	Ref.	Ref.
Living status	Alone	0.760 (0.570–1.013); 0.061	1.210 (0.901–1.625); 0.204	1.592 (1.056–2.406);**<0.001**
	With other people	0.682 (0.524–0.888);**0.004**	0.957 (0.738–1.241); 0.739	1.403 (0.969–2.032); 0.073
	With spouse only	Ref.	Ref.	Ref.
Working years [years]		1.007 (0.998–1.016); 0.118	1.012 (1.002–1.023);**0.022**	1.005 (0.991–1.019); 0.474
Financial situation of the household	Can afford when saving	0.670 (0.488–0.920);**0.013**	0.880 (0.653–1.185); 0.400	1.313 (0.849–2.025); 0.219
	Difficulties paying for food or clothes	0.613 (0.310–1.210); 0.158	0.615 (0.308–1.228); 0.168	1.004 (0.384–2.684); 0.993
	Can afford everything	Ref.	Ref.	Ref.
Cannot afford medications	Yes	0.921 (0.682–1.244); 0.590	0.589 (0.430–0.807);**<0.001**	0.640 (0.414–0.991);**0.044**
	No + No need	Ref.	Ref.	Ref.
Self-reported health status	Poor (0–3)	0.620 (0.423–0.911);**0.015**	0.811 (0.551–1.195); 0.290	1.307 (0.760–2.266); 0.335
	Fair (4–6)	0.733 (0.569–0.945);**0.016**	0.934 (0.740–1.178); 0.563	1.274 (0.903–1.797); 0.168
	Good (7–10)	Ref.	Ref.	Ref.

Univariate regression results. OR, odds ratio; CI, confidence interval; Ref, reference group.*p*-values are indicated in bold.

**Table 6:** Multivariate regression analysis of relationship between catarct treatment and the set of independent variables in PolSenior and PolSenior2 cohort.

Predictors	Categories	PolSenior cohort OR (95%CI); *p*-value	PolSenior 2 cohort OR (95%CI); *p*-value
Sex	Males	1.518 (1.169–1.972);**0.002**	1.490 (1.153–1.929);**0.002**
	Females	Ref.	Ref.
Age	>75	1.593 (1.196–2.129);**0.002**	1.625 (1.247–2.116);**<0.001**
	65–75	Ref.	Ref.
Cannot afford medications	Yes	-	0.605 (0.423–0.871);**0.006**
	No + No need	-	Ref.
Self-reported health status	Poor (0–3)	0.542 (0.346–0.841);**0.007**	-
	Fair (4–6)	0.709 (0.532–0.943);**0.018**	-
	Good (7–10)	Ref.	-

Multivariate regression results. OR, odds ratio; CI, confidence interval; Ref, reference group.*p*-values are indicated in bold.

The analysis of interactions between the cohorts and the risk factors showed that, over the decade, cataract surgery increased in the group of single-living and widowed individuals, while the lack of funds for medications was a significant factor reducing the chances for surgical management (Table 5).

## 4. Discussion

According to data from the National Health Fund (NHF) in Poland, 187,478 cataract surgeries were performed in 2013, and this number almost doubled (355,470) in 2019 (15). This increase was the result of changes in the organization and financing of cataract surgery. Our study also showed a significant increase in the percentage of cataract surgeries over the decade, i.e., from 46.5 to 67.9%. The increase was observed in each analysed age subgroup, both among women and men, and regardless of education or place of residence. An upward trend in cataract surgeries has also been shown in many other European countries (16), such as England (16, 17), and Germany (17).

The present study explained that despite a significant increase in cataract surgical treatment, there are widening inequalities in the distribution of this type of services. Of great concern is the fact that despite the higher declared prevalence of cataracts among women, they are actually less likely to undergo surgical treatment - this situation highlights inequalities in the distribution of cataract treatment.

Our study showed that the diagnostic rate of cataract in the population of older Poles did not increase over the decade, but significant differences were observed among women ≥75 years of age. This may be related to the improved access to ophthalmic care in this age group as well as growing awareness of cataract. In PolSenior2, women diagnosed with cataract were significantly less likely to receive surgical treatment compared to men. Previously conducted PolSenior also showed a difference in favour of men, but it did not reach statistical significance. The presented univariate and multivariate regression analyses confirmed in both editions of the study, that men with diagnosis of cataract were approximately 1.5 times more likely to undergo surgery than women. Gender inequality to the disadvantage of women in cataract surgery has also been observed in low-and middle-income countries (8, 18, 19). The accessibility of cataract surgery is diverse in high-income countries. In some countries, such as Sweden (20) and Spain (21), waiting times for cataract surgery are longer for women than for men, while in Canada women use ophthalmic services more often (22).

The reasons for the differences identified in our study may be multiple. Data from the PolSenior2 survey shows that women are more likely to be widowed and more likely to live alone (29.2 vs. 13.0%) (13). In general the burden of treatment, including cataract treatment for a one-person household, is higher. In addition, average pensions received in 2019 by women aged over 65 in Poland were 20% lower than men (23). Similarly, in the United States the most common reason for not having an eye-care visit among women aged 40 years and older with eye disease was cost-related (24). Less educational attainment also hinders the ability of women to obtain sufficient health care information (25, 26). Among Polsenior2 respondents with primary education, men were significantly more likely to undergo cataract surgery than women, and no differences were found among respondents with higher education.

The diagnosis of cataract increased with age in both study cohorts representative of the Polish population aged ≥65 years. The increasing prevalence of cataract with age is supported by other studies in the world (16, 27). In China, the prevalence of cataract was 6.7% among men aged 45–49 years and up to 73.0% among those aged 85–89 years, as well as 8.4 and 77.5%, respectively, among women (28). In PolSenior, no significant differences were found between the incidence of cataract and sex, while in PolSenior2 cataracts were more often reported by women. The inter-sex differences in PolSenior2 were particularly pronounced in the group with the lowest level of education and rural respondents. An international study investigating data from 1990 to 2015 revealed persistent global inter-sex differences in the prevalence of cataracts and found that older age and lower socio-economic status contribute to these differences (18). More common lens opacification in women may be associated with their longer life expectancy and decreased menopausal oestrogen levels (18, 29). In turn, a study which assessed the prevalence of age-related cataracts based on medical examination found no inter-sex differences (16).

PolSenior and PolSenior2 found that awareness of cataract diagnosis was significantly more often declared by inhabitants of large cities than by rural residents. This is probably related to poorer access to medical services in the countryside. Global research based on slit lamp examination for lens translucency did not show significant differences in the prevalence of cataracts between urban and rural regions (18, 20). The mean age at diagnosis in PolSenior was approximately 2 years higher than in PolSenior2 (74.2 vs. 72.1 years). The mean age at cataract diagnosis among Canadians with insurance covering routine annual ophthalmic examinations was 70.8 years, and 72.5 years among those without insurance, due to delayed access to an ophthalmologist (30). In our study, the earlier diagnosis among PolSenior2 respondents was probably associated with improved access to ophthalmic services. However, it cannot be ruled out that lifestyle changes in recent years and greater exposure to risk factors have translated into an increasingly earlier onset of senile cataracts.

Multiple barriers related to socio-economic status, sex, and perceived cost of ophthalmic care can limit patients’ access to specialist services (7). On the positive side, the situation of widowed and single people in Poland has improved over the last 10 years, and inequalities in access to cataract surgery among those with poorer self-rated health, poorer financial status, and those living with non-spouses, have been addressed. Many studies describe socio-economic status as a key determinant of the use of ophthalmic care services, which are used less as socio-economic disadvantages increase (7, 31). Unfortunately, the situation of people who declared lack of funds for medication worsened in the PolSenior2 cohort. Although intraocular lens surgery is fully reimbursed by the National Health Fund, patients have to cover the cost of postoperative anti-inflammatory eye drops and topical antibiotics, most of which are not reimbursed. Patients may therefore choose not to undergo the surgery both because of the perioperative costs, which are not limited to the purchase of medications, but also include commuting, the purchase of new glasses, as well as fear of possible complications. It should be emphasised that in contrast to the lack of funds for medications, the overall poor self-reported financial situation was not a factor limiting cataract surgery in PolSenior2. It can therefore be speculated that the lack of funds for medications may affect a group of people with multimorbidity, for whom medical expenses are a significant component of their household budget, and for whom treatment of visual impairment is not a health priority.

The data and conclusions of our study do not include the COVID-19 pandemic period, which had been associated with an approximately 30% decrease in medical services in 2020 compared to 2019 according to NHF data. Due to the pandemic, elective surgical procedures in Poland were suspended from March to May 2020, which again extended the waiting list. Therefore, we face a major challenge to re-establish an easy access to cataract surgery.

A strength of our study is the collection of data on a large, representative, community dwelling population aged 65 and older, with a high proportion of the oldest people. PolSenior projects are the largest studies of health status of older Poles. The almost identical sampling scheme and research methods in both PolSenior studies enabled precise comparisons between the two groups and provided additional evidence for the necessary change of health policies to eliminate health inequalities and improve access to surgical procedures. The limitations of our study are the following: obtaining data only on the basis of medical history, lack of data on waiting time between diagnosis and surgery as well as the source of funding and the type of lens implanted.

## 5. Conclusion

In 2009–2019, the prevalence of cataracts in the older Polish population remained unchanged, while the rates of cataract surgeries increased significantly over the decade. Despite the increase in declared cataracts among women, they still undergo operations less frequently than men - this situation shows the largest inequality in the distribution of cataract treatment services in Poland. Access to surgery among widowed and single-living people has improved while it has worsened among those declaring financial difficulties in purchasing medications. Cataracts are still a major medical and social challenge, and equalising the chances of its surgical treatment among selected groups of patients (women, people with lower socio-economic status) remains a challenge.

## Data availability statement

The original contributions presented in the study are included in the article/Supplementary material, further inquiries can be directed to the corresponding author.

## Ethics statement

The studies involving humans were approved by Bioethics Committee of the Medical University of Gdansk (NKBBN/257/2017). The studies were conducted in accordance with the local legislation and institutional requirements. The participants provided their written informed consent to participate in this study.

## Author contributions

NL and MM conceived the project and analysed results. AW performed the statistical analysis. NL wrote the manuscript. MM, HK-D, NL, KS, AL, DR, JJ-J reviewed and edited the manuscript. MM supervised the project. All authors contributed to the article and approved the submitted version.

## References

[1] GBD 2019 Blindness and Vision Impairment Collaborators, Vision Loss Expert Group of the Global Burden of Disease Study. Causes of blindness and vision impairment in 2020 and trends over 30 years, and prevalence of avoidable blindness in relation to VISION 2020: the right to sight: an analysis for the global burden of disease study. Lancet Glob Health. (2021) 9:144–60. doi: 10.1016/S2214-109X(20)30489-7 PMC7820391 33275949

[2] Cicinelli M Buchan JC Nicholson M Varadaraj V Khanna RC. Cataracts. Lancet. (2023) 401:377–89. doi: 10.1016/S0140-6736(22)01839-6, PMID: 36565712

[3] Han X Zou M Liu Z Sun Y Young CA Zheng D. Time trends and heterogeneity in the disease burden of visual impairment due to cataract, 1990–2019: a global analysis. Front Public Health. (2023) 11:1140533. doi: 10.3389/fpubh.2023.1140533, PMID: 37077196 PMC10106776

[4] Nowak MS Smigielski J. The prevalence of age-related eye diseases and cataract surgery among older adults in the City of Lodz, Poland. J Opt. (2015) 2015:605814:1–7. doi: 10.1155/2015/605814 PMC4350620 25789169

[5] Nowak MS Grabska-Liberek I Michalska-Małecka K Grzybowski A Kozioł M Niemczyk W. Incidence and characteristics of cataract surgery in Poland, during 2010-2015. Int J Environ Res Public Health. (2018) 15:435. doi: 10.3390/ijerph15030435 29498697 PMC5876980

[6] Tsou BC Vongsachang H Purt B Srikumaran D Justin GA Woreta FA. Cataract surgery numbers in U.S. ophthalmology residency programs: an ACGME case log analysis. Ophthalmic Epidemiol. (2022) 29:688–95. doi: 10.1080/09286586.2021.2015395, PMID: 34913813

[7] World Health Organization (2019). World report on vision. World Health Organization. Available at: https://apps.who.int/iris/handle/10665/328717. (Accessed September 25, 2023)

[8] Ramke J Zwi AB Palagyi A Blignault I Gilbert CE. Equity and blindness: closing evidence gaps to support universal eye health. Ophthalmic Epidemiol. (2015) 22:297–307. doi: 10.3109/09286586.2015.1077977, PMID: 26395657

[9] Hashemi H Pakzad R Khabazkhoob M. Decomposition of economic inequality in cataract surgery using Oaxaca blinder decomposition: Tehran geriatric eye study (TGES). Ophthalmic Epidemiol. (2022) 29:401–10. doi: 10.1080/09286586.2021.1946827, PMID: 34233572

[10] Khairallah M Kahloun R Bourne R Limburg H Flaxman SR Jonas JB. Number of people blind or visually impaired by cataract worldwide and in world regions, 1990 to 2010. Invest Ophthalmol Vis Sci. (2015) 56:6762–9. doi: 10.1167/iovs.15-17201 26567788

[11] Narodowy Instytut Zdrowia Publicznego PZH-Państwowy Instytut Badawczy (2021). Nierówności w zdrowiu i znaczenie ich pomiaru w ramach realizacji Narodowego Programu Zdrowia (NPZ). 1–8.

[12] Bledowski P Mossakowska M Chudek J Grodzicki T Milewicz A Szybalska A. Medical, psychological and socioeconomic aspects of aging in Poland: assumptions and objectives of the pol senior project. Exp Gerontol. (2011) 46:1003–9. doi: 10.1016/j.exger.2011.09.006, PMID: 21979452

[13] Wierucki Ł Kujawska-Danecka H Mossakowska M Grodzicki T Błędowski P Chudek J. Health status and its socio-economic covariates in the older population in Poland – the assumptions and methods of the nationwide, cross-sectional pol Senior2 survey. Arch Med Sci. (2022) 18:92–102. doi: 10.5114/aoms.2020.100898, PMID: 35154530 PMC8826695

[14] Szybalska A Broczek K Slusarczyk P Kozdron E Chudek J Puzianowska-Kuznicka M. Utilization of medical rehabilitation services among older poles: results of the pol senior study. Eur Geriatr Med. (2018) 9:669–77. doi: 10.1007/s41999-018-0077-8, PMID: 30294398 PMC6153710

[15] National Health Fund (NHF) (2021). National Health Fund (NHF) data. Available at: https://app.powerbi.com/view?r=eyJrIjoiYTUxNmIzZjItZDI0MC00ZGNlLWEyMTYtMTcxNjhhZTAwOTFlIiwidCI6IjJlNzcwYzE2LWMwNzEtNDA1Mi04MzdjLTU0NWJlZTBiMzQwYyIsImMiOjl9 [Accessed April 2021].

[16] Hashemi H Pakzad R Yekta A Aghamirsalim M Pakbin M Ramin S. Global and regional prevalence of age-related cataract: a comprehensive systematic review and meta-analysis. Eye (Lond). (2020) 34:1357–70. doi: 10.1038/s41433-020-0806-3, PMID: 32055021 PMC7376226

[17] Gianino MM Lenzi J Bonaudo M Fantini MP Siliquini R Ricciardi W. The switch between cataract surgical settings: evidence from a time series analysis across 20 EU countries. PLoS One. (2018) 13:0192620. doi: 10.1371/journal.pone.0192620, PMID: 29489834 PMC5831096

[18] Zetterberg M Celojevic D. Gender and cataract – the role of estrogen. Curr Eye Res. (2015) 40:176–90. doi: 10.3109/02713683.2014.898774, PMID: 24987869

[19] Lewallen S Mousa A Bassett K Courtright P. Cataract surgical coverage remains lower in women. Br J Ophthalmol. (2009) 93:295–8. doi: 10.1136/bjo.2008.140301, PMID: 19091848

[20] Smirthwaite G Lundström M Wijma B Lykke N Swahnberg K. Inequity in waiting for cataract surgery-an analysis of data from the Swedish National Cataract Register. Int J Equity Health. (2016) 15:10. doi: 10.1186/s12939-016-0302-3 26786522 PMC4717635

[21] Rius Ulldemolins A Benach J Guisasola L Artazcoz L. Why are there gender inequalities in visual impairment? Eur J Pub Health. (2019) 29:661–6. doi: 10.1093/eurpub/cky245 30500932

[22] Aljied R Aubin MJ Buhrmann R Sabeti S Freeman EE. Eye care utilization and its determinants in Canada. Can J Ophthalmol. (2018) 53:298–304. doi: 10.1016/j.jcjo.2018.01.021 29784169

[23] Eurostat data. (2021). Available at: https://ec.europa.eu/eurostat/web/products-eurostat-news/product/-/asset_publisher/VWJkHuaYvLIN/content/id/12278485/pop_up [Accessed February 2021].

[24] Centers for Disease Control and Prevention (CDC). Eye-care utilization among women aged > or =40 years with eye diseases--19 states, 2006-2008. MMWR Morb Mortal Wkly Rep. (2010) 59:588–91. 20489682

[25] Lou L Ye X Xu P Wang J Xu Y Jin K. Association of sex with the global burden of cataract. JAMA Ophthalmol. (2018) 136:116–21. doi: 10.1001/jamaophthalmol.2017.5668, PMID: 29242928 PMC5838943

[26] Klasen S Lamanna F. The impact of gender inequality in education and employment on economic growth: new evidence for a panel of countries. Fem Econ. (2009) 15:91–132. doi: 10.1080/13545700902893106

[27] Singh S Pardhan S Kulothungan V Swaminathan G Ravichandran JS Ganesan S. The prevalence and risk factors for cataract in rural and urban India. Indian J Ophthalmol. (2019) 67:477–83. doi: 10.4103/ijo.IJO_1127_17, PMID: 30900578 PMC6446631

[28] Song P Wang H Theodoratou E Chan KY Rudan I. The national and subnational prevalence of cataract and cataract blindness in China: a systematic review and meta-analysis. J Glob Health. (2018) 8:010804. doi: 10.7189/jogh.08.010804, PMID: 29977532 PMC6005639

[29] Garrigan H Ifantides C Prashanthi GS Das AV. Biogeographical and altitudinal distribution of cataract: a nine-year experience using electronic medical record-driven big data analytics in India. Ophthalmic Epidemiol. (2021) 28:392–9. doi: 10.1080/09286586.2020.1849741 33213243

[30] Jin YP Buys YM Xiong J Trope GE. Government-insured routine eye examinations and prevalence of nonrefractive vision problems among elderly. Can J Ophthalmol. (2013) 48:167–72. doi: 10.1016/j.jcjo.2013.01.002, PMID: 23769777

[31] Wang W Yan W Fotis K Prasad NM Lansingh VC Taylor HR. Cataract surgical rate and socioeconomics: a global study. Invest Ophthalmol Vis Sci. (2016) 57:5872–81. doi: 10.1167/iovs.16-19894, PMID: 27802517

