# Supplementary material for: Significant improvements in cataract treatment and persistent inequalities in access to cataract surgery among older Poles from 2009 to 2019: results of the PolSenior and PolSenior2 surveys
Source: Front Public Health. 2023 Oct 10;11:1201689. doi: 10.3389/fpubh.2023.1201689 (PMC10603189; doi:10.3389/fpubh.2023.1201689)
Supplement: Supplementary file 1 [file Table_1.DOCX]

Supplementary Material

Significant improvements in cataract treatment and persistent inequalities in access to cataract surgery among older Poles from 2009-2019: results of the PolSenior and PolSenior 2 surveys.

Natalia Lange* ^1^, Hanna Kujawska-Danecka ^2^, Adam Wyszomirski^3^, Klaudia Suligowska ^1,4^, Adrian Lange^1^, Dorota Raczyńska^5^, Justyna Jędrychowska-Jamborska^6^, Małgorzata Mossakowska^7^

^1^Department of Preventive Medicine and Education, Medical University of Gdańsk, Gdańsk, Poland

^2^Department of Internal Medicine, Connective Tissue Diseases and Geriatrics, Medical University of Gdańsk, Gdańsk, Poland

^3^Department of Adult Neurology, Faculty of Medicine Medical, University of Gdańsk, Gdańsk, Poland

^4^Department of Dental Techniques and Masticatory System Dysfunctions, Medical University of Gdańsk, Gdańsk, Poland

^5^Optimum Professor's Ophthalmology Centre, Gdańsk, Poland

^6^Clinical Unit , Department of Ophtalmology, Wojewódzki Hospital in Cracow, Cracow, Poland

^7^Aging and Longevity Strategic Project, International Institute of Molecular and Cell Biology, Warsaw, Poland

*** Correspondence:**

Natalia Lange, MD,

[kubiak@gumed.edu.pl](mailto:kubiak@gumed.edu.pl), Medical University of Gdańsk.

**Supplementary Table 1.** Comparison of patients with treated and untreated cataracts in PolSenior and PolSenior2. Unweighted data.

| **PolSenior** | | | **PolSenior2** | | |  |
| --- | --- | --- | --- | --- | --- | --- |
|  | **Treated  cataract**  **N = 600** | **Untreated  cataract**  **N = 684** | **P- value** | **Treated  cataract**  **N = 1140** | **Untreated cataract**  **N = 461** | **P-value** |
| **Age at diagnosis** [years]  **Females**  Mean (SD)  Median (Q1, Q3) | 73.7 (10.7)  75 (68, 81) | 73.6 (9.9)  74 (68, 80) | 0.617 | 71.9 (11.2)  74 (67, 79) | 71.3 (13.8)  72 (66, 80) | 0.590 |
| **Age at diagnosis** [years]  **Males**  Mean (SD)  Median (Q1, Q3) | 75.0 (10.6)  76 (69, 82) | 74.3 (13.3)  75 (70, 82) | 0.952 | 72.8 (10.9)  74 (67,5, 80) | 73.2 (14.3)  74 (69, 82) | 0.191 |
| **Total**  Mean (SD)  Median (Q1, Q3) | 74.4 (10.7)  75 (69, 82) | 73.9 (11.4)  75 (68, 82) | 0.486 | 72.3 (11.1)  74.0 (67, 79,5) | 72.0 (14.0)  73.0(67, 80) | 0.800 |
| **Age** [years]  Mean (SD)  Median (Q1, Q3) | 82.4 (8.0)  83.0 (77, 88) | 81.4 (8.6)  81.0 (74, 88) | 0. 041 | 81.2 (7.8)  81.0 (75,8, 87) | 79.6 (8.3)  80.0 (73, 86) | <0.001 |
| **Age**  65-75  >75 | 134 (22.3%)  466 (77.7%) | 206 (30.1%)  478 (69.9%) | 0.002 | 285 (25.0%)  855 (75%) | 161 (34.9%)  300 (65.1%) | <0.001 |
| **Females** [N; %]  Males [N;%] | 283 (47.2%)  317 (52.8%) | 412 (60.2%)  272 (39.8%) | <0.001 | 618 (54.2%)  522 (45.8%) | 297 (64.4%)  164 (35.6%) | <0.001 |
| **Place of residence** [N; %]  Rural  Urban | 195 (32.5%)  405 (67.5%) | 205 (30.0%)  479 (70.0%) | 0.329 | 322 (28.2%)  818 (71.8%) | 149 (32.3%)  312 (67.7%) | 0.105 |
| **Marital status** [N; %]  Unmarried/Divorced  or separated  Married  Widowed | 18 (3.1%)  281 (48.9%)  276 (48.0%) | 23 (3.5%)  267 (40.6%)  367 (55.9%) | 0.015 | 51 (4.6%)  546 (48.9%)  519 (46.5%) | 22 (5.0%)  223 (50.5%)  197 (44.6%) | 0.771 |
| **Living status** [N; %]  Alone  With spouse only  With other people | 130 (23.0%)  273 (48.2%)  163 (28.8%) | 161 (25.0%)  257 (40.0%)  225 (35.0%) | 0.012 | 293 (27.5%)  379 (35.6%)  393 (36.9%) | 99 (23.5%)  155 (36.7%)  168 (39.8%) | 0.263 |
| **Education** [N; %]  Primary or incomplete primary  Basic vocational  Middle, secondary or post-secondary  Higher | 289 (50.5%)  66 (11.5%)  166 (29.0%)  51 (8.9%) | 355 (55.5%)  65 (10,2%)  166 (25.9%)  54 (8.4%) | 0.384 | 401 (35.8%)  209 (18.6%)  350 (31.2%)  161 (14.4%) | 167 (37,2%)  80 (17.8%)  146 (32.5%)  56 (12.5%) | 0.730 |
| **Years of education** [years]  Mean (SD);  Median (Q1, Q3) | 8.9 (3.8)  8 (7, 11) | 8.6 (3.8)  7 (7,11) | 0.187 | 10.4 (3.9)  10 (7,13) | 10.6 (4.2)  10 (7,13) | 0.640 |
| **Type of job**  **(now or in the past)** [N; %]  Manual/farming  Intellectual  Other  None | 317 (55.6%)  193 (33.9%)  40 (7.0%)  20 (3.5%) | 373 (57.6%)  194 (29.9%)  50 (7.7%)  31 (4.8%) | 0.384 | 575 (52.0%)  367 (33.2%)  83 (7.5%)  80 (7.2%) | 226 (51.4%)  141 (32%)  41 (9.3%)  32 (7.3%) | 0.697 |
| **Years of work**  Mean (SD);  Median (Q1, Q3) | 35.8 (12.7)  37.0 (30, 43) | 34.6 (13.8)  35.5 (28, 42,8) | 0.177 | 36.2 (11.0)  38, 0 (30,42) | 34.7 (11.8)  35, 0 (30,42) | 0.013 |
| **The frequency of GP appointments** [N; %]  More than once a year  Once a year or less | 500 (89.4%)  59 (10.6%) | 554 (87.9%)  76 (12.1%) | 0.413 | 900 (94.1%)  56 (5.9%) | 364 (95.8%)  16 (4.2%) | 0.229 |
| **Ophthalmology appointment  in the last 12 months** [N; %] | – | – | – | 317 (28.7%) | 135 (30.4%) | 0.508 |
| **Financial situation  of the household** [N; %]  Can afford everything  Can afford when saving  Difficulties paying for food  or clothes | 106 (20.1%)  404 (76.5%)  18 (3.4%) | 83 (14.4%)  472 (81.7%)  23 (4.0%) | 0.040 | 200 (18.2%)  875 (79.5%)  26 (2.4%) | 71 (16.2%)  353 (80.4%)  15 (3.4%) | 0.358 |
| **Cannot afford**  **for medications** [N; %]  No  Yes  No need, not sure | 469 (81.6%)  93 (16.2%)  13 (2.3%) | 519 (79.4%)  113 (17.3%)  22 (3.4%) | 0.424 | 970 (87.4%)  116 (10.5%)  24 (2.2%) | 360 (80.4%)  74 (16.5%)  14 (3.1%) | 0.002 |
| **Cannot afford  for appointments**  No  Yes  No need, not sure | 337 (59.1%)  41 (7.2%)  192 (33.7%) | 368 (56.7%)  37 (5.7%)  244 (37.6%) | 0.264 | 671 (60.6%)  141 (12.7%)  296 (26.7%) | 265 (59.2%)  66 (14.7%)  117 (26.1%) | 0.573 |
| **Self-reported health status** [N; %]  Poor (0-3)  Fair (4-6)  Good (7-10) | 61 (11.0%)  294 (53%)  200 (36.0%) | 88 (14.1%)  359 (57.3%)  179 (28.6%) | 0.016 | 101 (9.0%)  564 (50.1%)  461 (40.9%) | 47 (10.5%)  228 (50.8%)  174 (38.8%) | 0.555 |
| **Are you able to watch TV?**  Yes, with glasses  Yes, without glasses  No | 228 (40.0%)  309 (54.2%)  33 (5.8%) | 296 (46.6%)  288 (45.4%)  51 (8.0%) | 0.007 | 382 (35.2%)  655 (60.4%)  47 (4.3%) | 231 (53.7%)  188 (43.7%)  11 (2.6%) | <0.001 |
| **Spending free time during  the last 12 months** [N;%]  Reading books or newspapers | 417 (71.9%) | 448 (68.1%) | 0.145 | 862 (77.0%) | 330 (73.7%) | 0.166 |
| Watching TV, movies | 533 (91.9%) | 589 (89.5%) | 0.151 | 1044 (93.4%) | 411 (91.7%) | 0.253 |
| Using the Internet | 24 (4.1%) | 29 (4.4%) | 0.815 | 236 (21.3%) | 108 (24.3%) | 0.196 |
| Physical activities  (e.g. going for walks) | 373 (64.3%) | 414 (62.9%) | 0.611 | 758 (67.8%) | 296 (66.2%) | 0.547 |
| **Need of regular help  from other people?**  YES  NO | 263 (45.8%)  311 (54.2%) | 327 (50.2%)  324 (49.8%) | 0.123 | 470 (42.7%)  635 (57.5%) | 188 (42.2%)  258 (57.8%) | 0.891 |

**Abbreviations:** SD, standard deviation; IQR, interquartile range
